# The Effect of Repass Treatment on the Mechanical Properties and Microstructure of CF/PEKK Thermoplastic Composite Laminates Manufactured Using Laser-Assisted Automated Fiber Placement

**DOI:** 10.3390/polym17010073

**Published:** 2024-12-30

**Authors:** Xi Zhang, Xiaodong He, Hualian Li, Shenglai Wang

**Affiliations:** 1 College of Mechanical Engineering, Inner Mongolia University of Technology, Hohhot 010051, China; 20221800015@imut.edu.cn (X.Z.); hualianlihzhe@126.com (H.L.); 2 Inner Mongolia Aerospace Honggang Machinery Co., Ltd., Hohhot 010070, China; 16647160519@163.com

**Keywords:** CF/PEKK, automated fiber placement (AFP), mechanical properties, repass treatment

## Abstract

The emerging thermoplastic composite material PEKK exhibits superior thermal stability compared to PEEK. In this work, CF/PEKK laminates were fabricated using laser-assisted heating in AFP, and the effects of repass treatment on the mechanical properties and microstructure of the laminates were compared. The results show that after a single repass treatment, the tensile strength of the laminates increased by 28.39%, while the interlaminar shear strength increased by 11.9%, likely due to the distinct load-bearing components under the two loading conditions. Additionally, the repass treatment significantly improves the fiber/resin interface and surface roughness of the laminates.

## 1. Introduction

**Figure 1:**
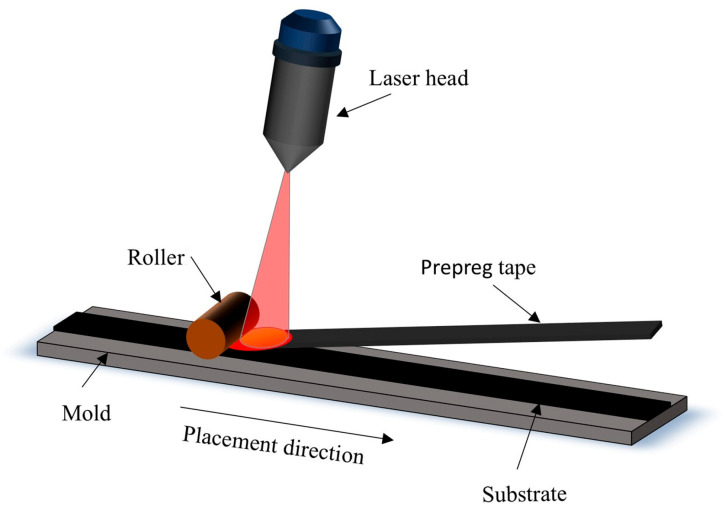
Schematic diagram of automatic placement of PEKK laminates.

The composition of thermoplastic composites has become increasingly complex, accompanied by a steady expansion of their applications across various sectors, particularly in the aerospace industry [1]. I. Martín et al. [2] employed the in situ consolidation of thermoplastic composites, along with the co-consolidation of aircraft skins and stringers, to enhance fuselage structures. Traditionally, methods such as filament winding and autoclave molding were employed for composite material fabrication; however, these techniques are characterized by low efficiency and high production costs [3,4]. automated fiber placement (AFP) technology, renowned for its high deposition efficiency and material utilization, has been increasingly adopted in a wide range of applications [5,6,7]. Improving the quality of thermoplastic composite laminates produced by AFP remains a critical challenge. As shown in Figure 1, AFP involves loading segmented prepreg tapes onto spools, with an industrial robot programmed to execute specific placement paths. The placement head, mounted on the robot, performs functions such as heating, clamping, cutting, and repositioning to form the laminates. Numerous factors influence the mechanical properties of AFP-manufactured laminates, including placement speed, placement temperature, and compaction roller pressure [8,9,10,11,12]. Many researchers have conducted studies and analyses on these parameters. For instance, Ziang Jin et al. [13] investigated the temperature field generated by a slit-shaped nozzle using a hot gas torch as the heat source, demonstrating that it provides a more uniform temperature distribution compared to a circular nozzle. Chen Liu et al. [14] specifically analyzed the effect of pressure on a Mode I interlaminar fracture in PEEK (poly ether ketone ketone) laminates, demonstrating that high pressure improves the resin/fiber interface and inhibits crack propagation in the resin. Chenping Zhang et al. [15] revealed that higher heating temperatures, increased compaction roller pressures, and lower placement speeds contribute to warping deformation in curved laminates. The strength of AFP-manufactured laminates exhibits a significant disparity compared to autoclave-consolidated products; a difference primarily attributed to the unique processing characteristics of AFP. Emad Pourahmadi et al. [16] pointed out that porosity and fiber alignment significantly affect the stress distribution and concentration within the matrix thereby influencing the mechanical properties of the material. In AFP processing, the heating and cooling times for the tape, as well as the duration during which the compaction roller applies consolidation force, are notably brief. The former results in reduced crystallinity, while the latter shortens the interlayer intimate contact time, leading to increased porosity. Both factors contribute to a reduction in the interlaminar shear strength (ILSS) of the laminate [10,17]. Experimental results from Emad Pourahmadi et al. [18] demonstrated that the interlaminar shear strength (ILSS) of AFP-manufactured CF/PEEK (carbon fiber/poly ether ketone ketone) products is 37% lower compared to those produced using autoclave molding. Shadmeri et al. [1] employed a hot gas torch for the repass treatment of CF/PEEK laminates, which reduced surface roughness but also slightly decreased crystallinity. Angeliki Chanteli et al. [19] combined different tape placement directions with repass treatment angles, confirming that the repass treatment enhances the open-hole compression (OHC) and in-plane shear (IPS) properties of the laminates. Qiuyu M. et al. [20] fabricated laminates by alternating CF/PEEK with PEEK films, followed by repass treatment, which significantly reduced porosity and improved the ILSS of the laminates. However, studies on the effects of repass treatment on the mechanical properties and microstructure of laser-assisted manufactured CF/PEKK laminates remain scarce.

As an emerging crystalline polymer, PEKK exhibits superior thermal stability compared to PEEK [21,22]. The limited availability of literature on PEKK means that studies related to PEEK and CF/PEEK can be referenced for broader comparisons. In recent years, laser heating has been increasingly adopted due to its ability to overcome all the shortcomings of hot gas torches. However, it comes with its own drawbacks, such as high cost and the risk of thermal degradation. Wei Jiang et al. [23] proposed a method to reduce lateral scattering of the laser during the AFP process thereby enhancing laser absorption and heating efficiency. PEKK has a lower crystallinity compared to PEEK, making it less affected by the cooling process. In the context of laser-assisted AFP, where the tape cooling time is brief, this property makes PEKK more suitable for processing. Therefore, laser heating was selected in this study.

This study investigates the effects of repass treatment on the strength performance and microstructure of CF/PEKK laminates manufactured using laser-assisted AFP. Four different laminate manufacturing strategies were examined: laminates without repass treatment, laminates subjected to one repass treatment parallel to the fiber direction, two repass treatments parallel to the fiber direction, and three repass treatments parallel to the fiber direction. Samples were fabricated using the aforementioned methods and subjected to ILSS and tensile strength tests to evaluate the impact of reprocessing on mechanical properties. In addition, a surface roughness tester was used to measure the changes in surface roughness of the laminates induced by laser reprocessing. Finally, scanning electron microscopy was used to observe cross-sections of the samples, providing insights into the effects of repass treatment on the internal structural characteristics of the laminates.

## 2. Experimental Methods

### 2.1. Material and Equipment

**Figure 2:**
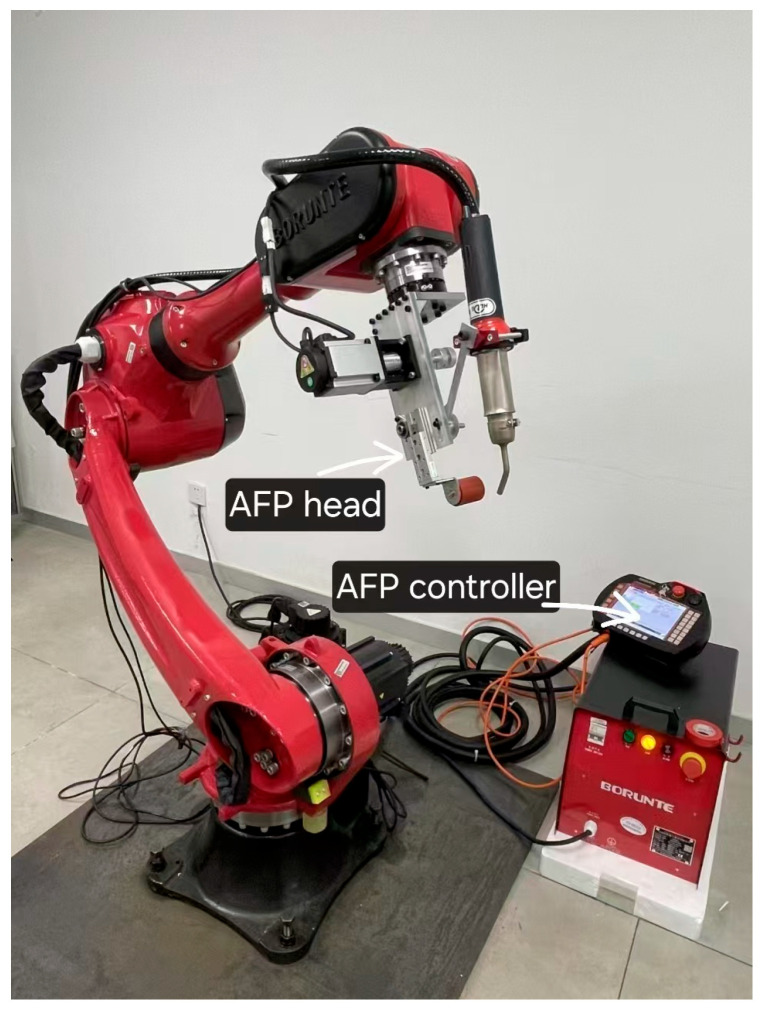
AFP devices.

The CF/PEKK prepreg tape used in this study had a resin mass fraction of 31%, a thickness of 0.19 mm, a width of 12 mm, and a density of 1.58 g/cm^3^ (supplied by Jiangsu Bi-gold New Material Stock Co., Ltd., Jurong City, China). The laminates were fabricated using an AFP head mounted on a BORUNTE six-axis robot (Figure 2). The AFP head consisted of the following components: (a) a 100 W pulsed laser heat source with a wavelength of 900 nm and a beam width of 8 mm; (b) a tape conveying, guiding, and cutting system; and (c) a consolidation roller made of silicone material with a diameter of 32 mm and a length of 40 mm, driven by a cylinder-guided rail system.

### 2.2. Manufacturing of Laminates

Shadmeri et al. [1] proposed that repass treatment refers to the application of heat and pressure to the substrate layers using the AFP head without adding new material. Laser heating involves the irradiation of the material’s surface with laser pulses, generating heat in the illuminated area. The heat is then transferred from the surface to the interior of the material through mechanisms such as thermal diffusion, convection, and conduction, achieving the desired heating effect.

**Figure 3:**
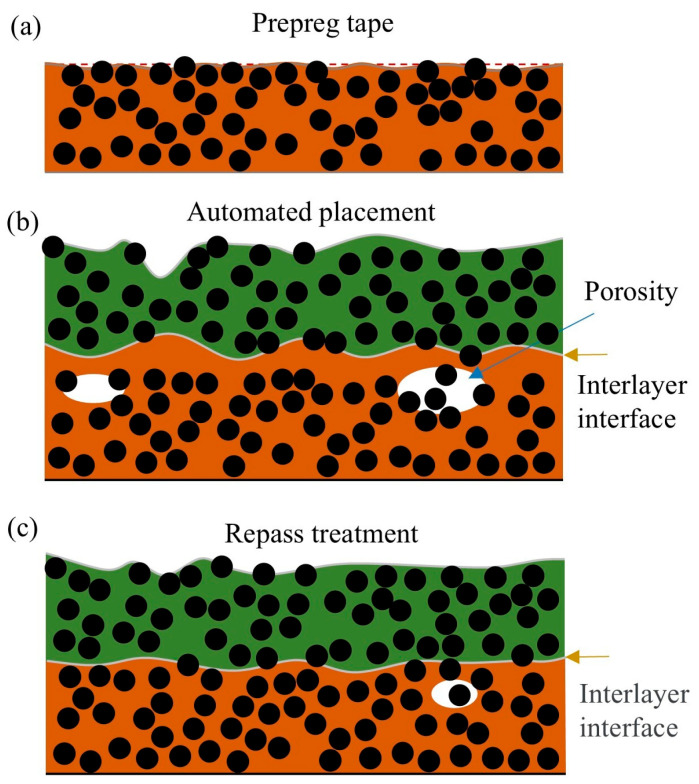
Schematic diagram of effect of repass treatment on quality of laminates: a-prepreg tape, b-automated placement, c-repass treatment.

Taking CF/PEKK as an example, its long-chain structure, composed of numerous ether and ketone bonds, is wrapped around the carbon fibers. The only interactions between the chains are van der Waals forces and chain entanglements. In Figure 3a, at room temperature, molecular motion is minimal and van der Waals forces maintain the material in a solid state. Under these conditions, the surface of the prepreg tape appears smooth and flat. In Figure 3b, the surface of the laminate appears rough, with exposed fibers, irregular interlayer interfaces, and numerous voids present. This is because, during the placement process, the differences in thermal expansion coefficients and thermal conductivities between the resin and fibers cause them to heat at different rates under laser irradiation. Upon laser irradiation, the resin undergoes a significant temperature change, transitioning from a solid to a flowing state. In contrast, the fibers can only absorb heat from the heated resin, resulting in minimal volumetric change. At this stage, the compaction roller applies pressure, causing the resin to flow and fill the voids between the fibers. However, to prevent thermal degradation of PEKK, the processing parameters are subject to certain limitations, which restrict the resin from fully flowing. In Figure 3c, the repass treatment improves surface roughness, reduces porosity, and alleviates defects and stress concentrations at the resin/fiber interface. These improvements collectively enhance the mechanical properties of the laminate.

For simplicity, the four groups of laminates are designated as NR, 1R, 2R, and 3R. NR represents laminates without repass treatment, while 1R, 2R, and 3R correspond to laminates subjected to one, two, and three repass treatments, respectively, parallel to the fiber direction. The four groups of laminates were used as references to study the effects of reprocessing on fiber/resin distribution, surface roughness, ILSS, and tensile strength.

The quality of the laminates is closely related to the optimization of processing parameters. The processing parameters include (a) laser power, (b) placement speed, (c) compaction roller pressure, and (d) compaction roller material. As shown in Table 1, these parameters were applied to the four groups of laminates. These parameters were determined with reference to the recommendations provided by the PEKK material manufacturer.

### 2.3. Physical Properties Test

Surface roughness testing of the laminates was conducted using a Bruker Npflex 3D optical profiler, with 10 samples measured for each group. A probe was used to perform measurements at two different regions on each sample, and the arithmetic mean deviation ($R_{a}$) was calculated.

The ILSS test was conducted according to the ISO 14130:1997 standard [24], with sample dimensions of 20 mm × 10 mm × 2 mm and six samples per group. The loading displacement rate was set to 1 mm/min. The ILSS was calculated using the following formula:

(1) $$\tau_{m}=\frac{3}{4}\times \frac{F}{bh}$$

where $\tau_{m}$ represents the ILSS value (MPa), *F* is the maximum load (N), *b* is the sample width (mm), and *h* is the sample thickness (mm).

The tensile strength test was conducted in accordance with the ISO 527-4:1997 standard [25]. The samples were dumbbell-shaped with dimensions of 180 mm × 10 mm × 2 mm, where 10 mm represents the width of the parallel section in the middle of the dumbbell, and the ends were 20 mm wide. Six samples were tested per group. The tensile strength was calculated using the following formula:

(2) $$\sigma_{t}=\frac{F}{A}$$

where $\sigma_{t}$ represents the tensile strength value (MPa), *F* represents the maximum tensile force experienced by the material during the test (N), and *A* denotes the cross-sectional area of the specimen (mm²).

In addition, the cross-sections of the laminates were polished and observed using an optical microscope to examine the fiber/resin distribution characteristics. The failure modes of the ILSS and tensile strength test specimens were also analyzed.

## 3. Results and Discussion

### 3.1. Surface Roughness Measurements

**Figure 4:**
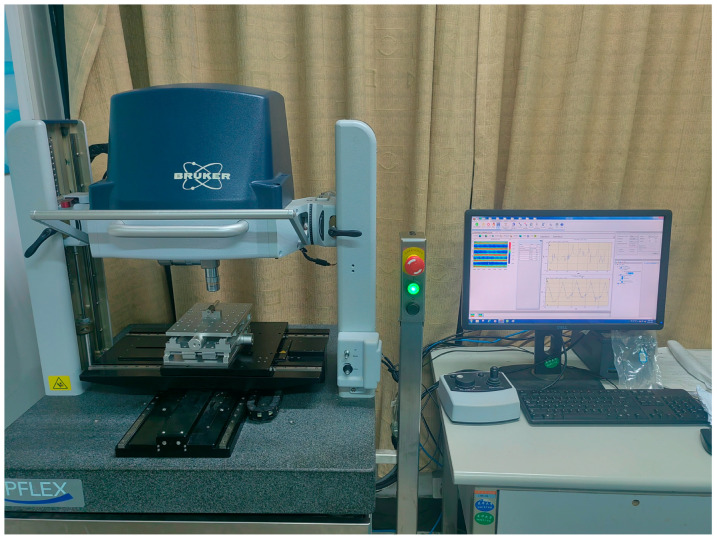
Surface roughness equipment.

**Figure 5:**
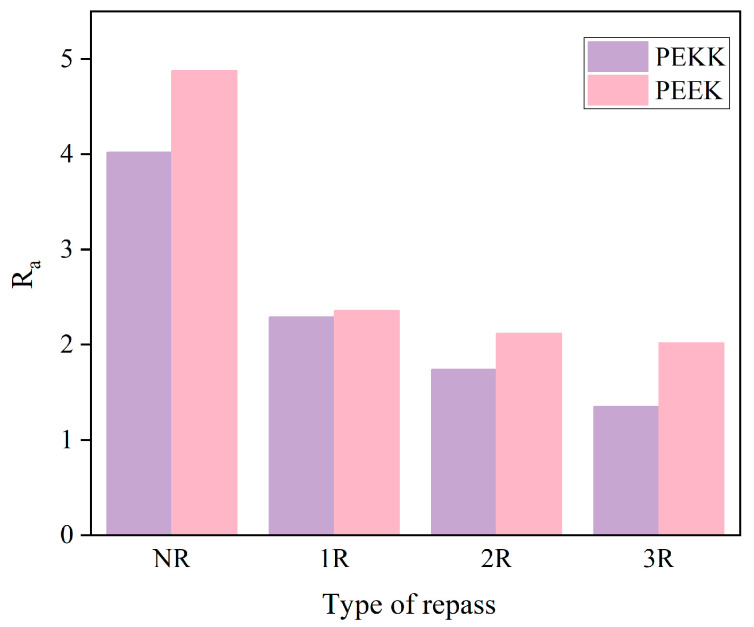
Surface roughness measurement.

**Figure 6:**
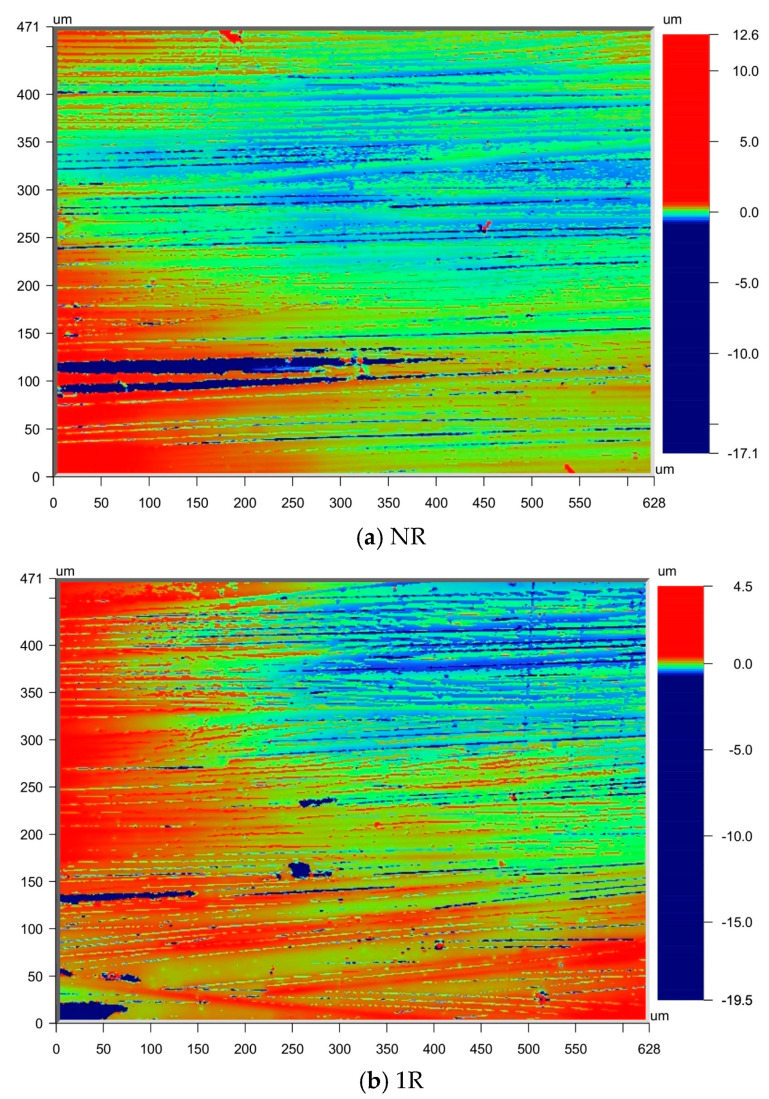
Surface roughness measurement image.

The surface roughness of the specimens was measured using the Bruker Npflex 3D optical profiler(Bruker Corporation, Billerica, Massachusetts, USA), as shown in Figure 4. Table 2 and Figure 5 present the surface roughness values of the laminates. From the surface roughness values of the samples, the overall trends for CF/PEKK and CF/PEEK are similar. The 1R process significantly improved surface roughness, while 2R caused a slight increase in roughness. The results for 3R were comparable to those of 2R. Figure 6 shows a comparison of the surface roughness measurements between the NR and 1R samples. It is evident that the range of surface texture fluctuations in 1R is smaller and closer to the average value, further confirming that reprocessing improves the smoothness of the laminate surface. The results indicate that repass treatment has a significant effect on improving surface roughness, with the improvement ranging from 18.2% to 42.6%. Under the same processing conditions, the $R_{a}$ (Arithmetic Average Roughness) value of PEKK is slightly lower than that of PEEK. This is likely due to PEKK’s higher ketone content, as ketone bonds exhibit better thermal stability than ether bonds. This enhanced thermal stability can reduce thermal deformation during processing, resulting in a smoother surface. In addition, factors such as the number of laminate layers and the manufacturing process of the prepreg tape also influence the surface roughness results. Further research is needed to determine the exact mechanisms behind the improvement in surface roughness.

### 3.2. Microstructure

**Figure 7:**
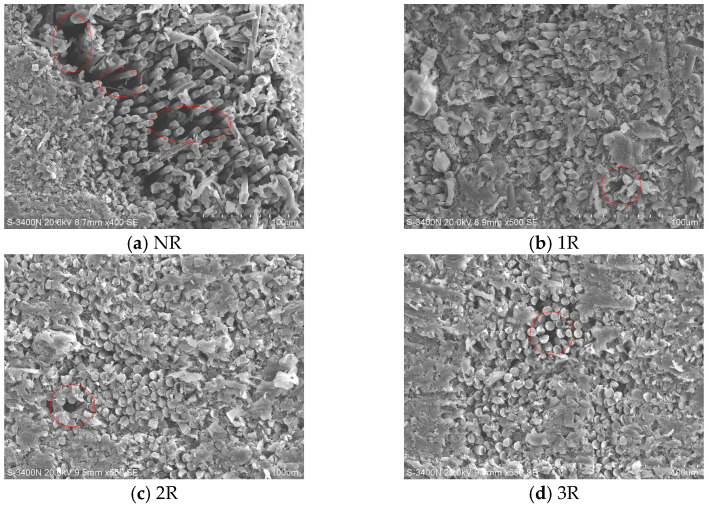
Microstructure.

Since PEKK resin is almost non-conductive, the samples were first sprayed with gold and then attached to conductive adhesive before being imaged using a Hitachi S-3400N scanning electron microscope. Figure 7 illustrates the microstructure of the laminates from each group. The microscopic images clearly show that all laminates exhibit good adhesion. However, noticeable voids are present between the layers of the laminates without repass treatment (Figure 7a). This is due to the short heating time and limited interlayer intimate contact time, which prevent the resin from fully flowing and adequately bonding with the fibers. After undergoing one, two, and three repass treatments, which provided additional heat and pressure, the voids were significantly reduced (Figure 7b–d).

Equation (3) is the tight contact model proposed by Lee W I et al. [26]. In this model, $D_{ic}$ represents the degree of intimate contact; $w_{0}$, $a_{0}$, and $b_{0}$ are the geometric parameters of the tape surface; $t_{c}$ is the duration of temperature and pressure applied during processing; $P_{app}$ is the applied pressure; and $u_{mf}$ is the viscosity. Repass treatment can be regarded as the reapplication of heat and pressure, which effectively increases the upper limit of $t_{c}$. Therefore, repass treatment results in a tighter interlaminar bond in the laminate and a more uniform fiber/resin distribution.

(3) $$D_{ic}=\frac{1}{1+\frac{w_{0}}{b_{0}}}{\left[1+5(1+\frac{w_{0}}{b_{0}}){\left(\frac{a_{0}}{b_{0}}\right)}^{2}\int_{0}^{t_{c}}\frac{P_{app}}{u_{mf}}dt\right]}^{\frac{1}{5}}$$

### 3.3. Interlaminar Shear Strength

**Figure 8:**
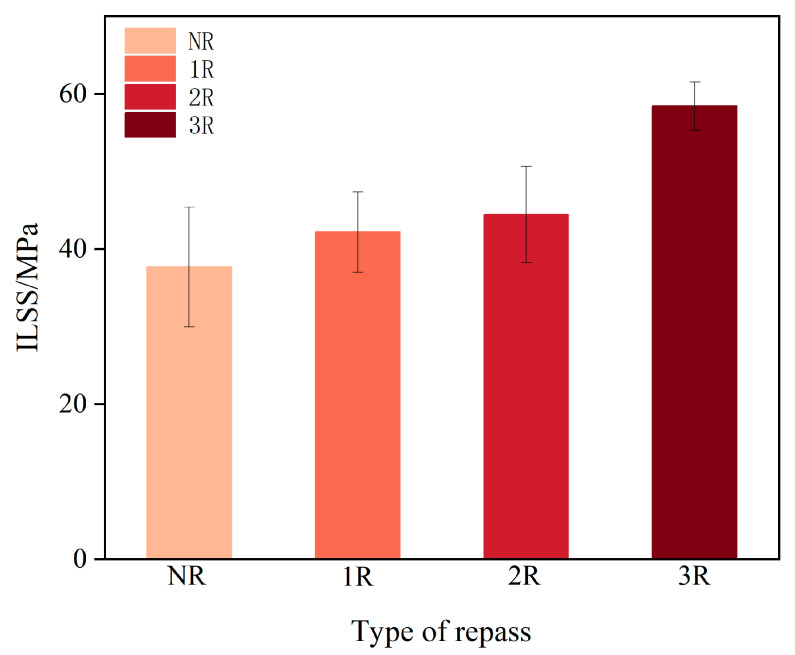
ILSS measurements.

Table 3 and Figure 8 present the ILSS values of the laminates from each group. Compared to NR, samples 1R, 2R, and 3R increased the ILSS by 11.9%, 18%, and 55%, respectively. As seen in Table 3 and Figure 8, the 3R samples exhibit the greatest increase in ILSS, along with the smallest standard deviation. However, this is also partly influenced by the temperature and pressure during placement and repass treatment, as well as the manufacturing parameters of the PEKK prepreg tape.

**Figure 9:**
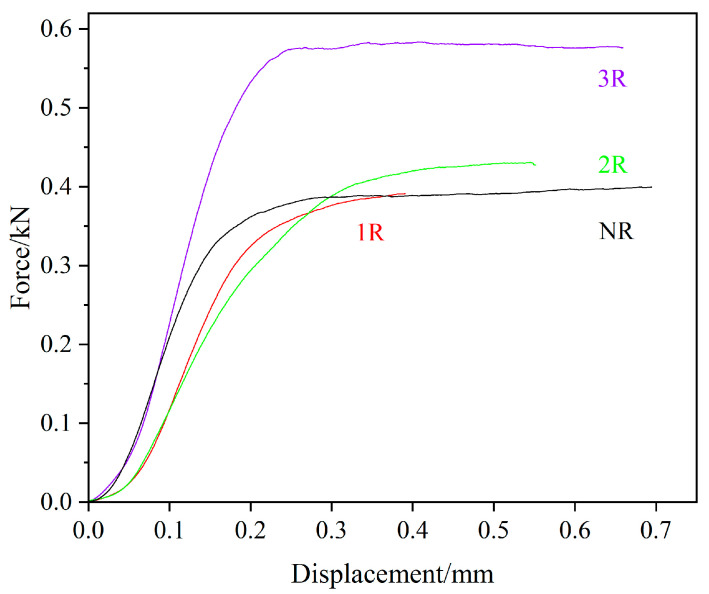
Force–displacement curve.

Figure 9 shows the force–displacement curves. The peak force gradually increases, indicating that repass treatment improves the interfacial adhesion of the laminates. The peak displacement shows minimal variation, and the overall trend of the curves remains similar, suggesting that the repass treatment primarily enhances interfacial adhesion while having a limited impact on the ductility of the material.

**Figure 10:**
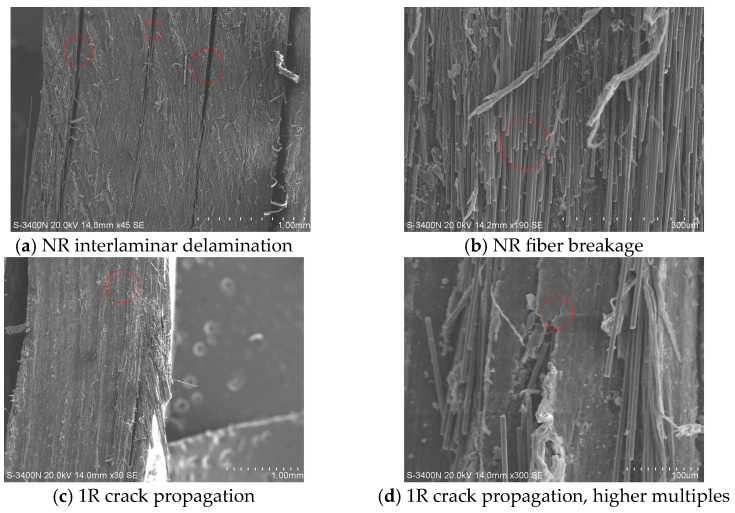
ILSS failure mode.

The SEM images of the ILSS failed specimens are shown in Figure 10. The NR specimen (Figure 10a) exhibits evident delamination, with the sample crushed at the loading point, followed by fiber deformation and fracture. Figure 10b presents a more detailed view of the NR specimen, revealing numerous fiber pull-outs on the sample surface. This indicates poor interfacial adhesion, where fibers lose their fixation within the resin, leading to fractures and even partial fiber lifting. The failed 1R specimen (Figure 10c,d) shows a tighter interlayer interface with no obvious delamination. Small cracks are present in the resin but are suppressed, and the resin is uniformly distributed. The improved interfacial bonding results in better overall performance of the laminate.

### 3.4. Tensile Strength

**Figure 11:**
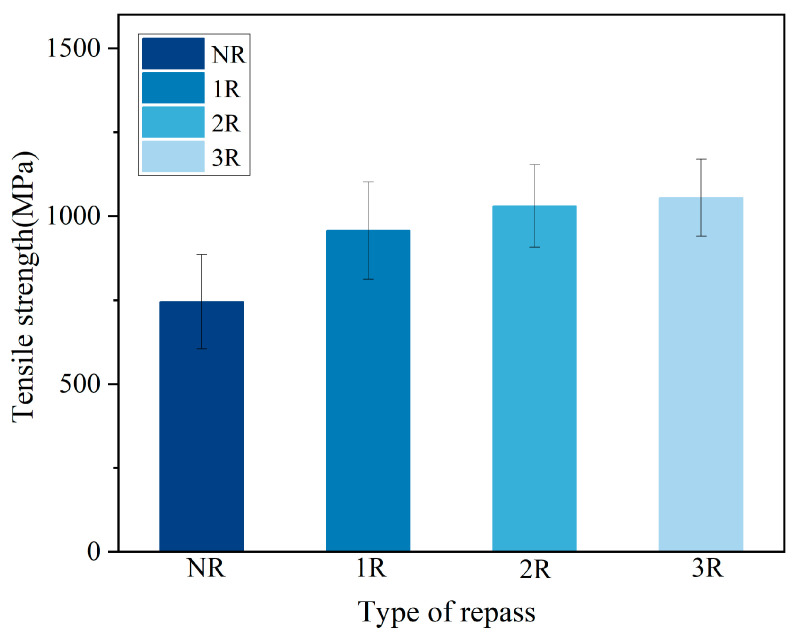
Tensile strength measurements.

Table 4 and Figure 11 present the tensile strength of the samples. 1R, 2R, and 3R increased the tensile strength of the laminates by 28.39%, 38.22%, and 41.56%, respectively. Using the 1R specimen as a reference, repass treatment resulted in a relatively small improvement in ILSS but a significant increase in tensile strength. This could be attributed to the following two reasons: (1) Repass treatment can reduce crystallinity. Although the final result shows an increase in ILSS, changes in crystallinity still have an impact on ILSS. (2) According to the rule of mixtures for composites, tensile strength primarily depends on the strength of the fibers rather than the strength of the resin. The decrease in resin crystallinity has a negligible effect on tensile strength. However, the heat and pressure generated by repass treatment allow the resin to flow more thoroughly, leading to tighter bonding between the resin and fibers and a reduction in porosity. The fiber/resin interface plays a crucial role in the mechanical properties of the composite, as it is responsible for transferring stress from the resin to the fibers. Repass treatment makes the interfaces between adjacent layers less distinguishable, increasing the number of fibers that simultaneously share the tensile load. Therefore, repass treatment has a significant impact on tensile strength.

**Figure 12:**
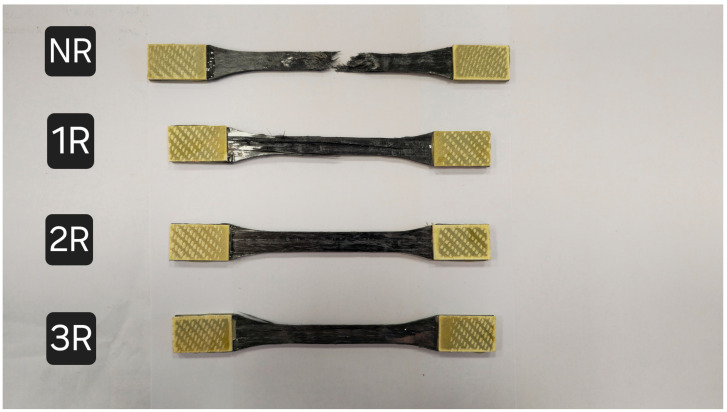
Tensile strength failure modes.

The failure modes of the tensile test specimens are shown in Figure 12. In the NR specimens, the surface fibers lose adhesion to the resin, followed by fiber fracture and lifting, and ultimately being pulled apart. The fracture surface exhibits a stepped pattern after failure. In the 1R specimens, a small number of fibers on the surface are lifted, followed by the appearance of an almost through-thickness crack in the center of the specimen. The failure modes of the 2R and 3R specimens are similar, with cracks appearing only in the middle of the specimen. This indicates that repass treatment has a significant impact on improving bonding strength at the fiber/resin interface.

## 4. Conclusions

This study investigated the effects of repass treatment under laser assistance on the mechanical properties, surface quality, and microstructure of CF/PEKK laminates. The main conclusions are as follows:

(1) Repass treatment significantly improves the surface roughness of PEKK laminates, with the $R_{a}$ value decreasing from 4.02 µm to 1.35 µm. Repass treatment mitigates issues such as fiber pull-out, resin residue, and voids generated during the initial placement, resulting in a smoother and more even surface.

(2) SEM images clearly demonstrate that repass treatment has a significant impact on the fiber/resin interface. Repass treatment allows the resin to flow sufficiently, reducing voids and enhancing interfacial bonding strength.

(3) One repass treatment increased the ILSS and tensile strength of the laminates by 11.9% and 28.39%, respectively. The difference in the improvement magnitude is primarily due to the fact that different components bear the load under the two types of loading conditions. Overall, repass treatment improves the mechanical properties of laminates. Considering factors such as processing efficiency, cost, and environmental sustainability, a single repass treatment is generally sufficient to meet the mechanical performance requirements. If high surface roughness is the primary requirement, a single repass treatment can be applied exclusively to the final layer.

(4) Under different testing conditions, CF/PEKK exhibits various failure modes. Macroscopic failure is mainly characterized by fiber breakage, interlaminar delamination, and crack propagation, while microscopic failure primarily manifests as damage to the fiber/matrix interface, matrix cracking, and the early stages of fiber fracture. Overall, repass treatment significantly enhanced the mechanical performance of CF/PEKK composites, especially in terms of tensile strength and interlaminar shear strength.

In summary, repass treatment improves the surface roughness, interlaminar shear strength, tensile strength, and fiber/resin interface of CF/PEKK laminates. However, whether the repass treatment temperature, fiber volume fraction of the prepreg, and the number of layers significantly affect the final results requires further investigation. Additionally, in the temperate continental climate where the authors are based, with significant variations in temperature and humidity over time, further observation and research are needed to determine whether the improvements brought by repass treatment are sustained or degrade under long-term environmental conditions.

## Figures and Tables

**Table 1:** Process parameters. Values in parentheses show laser power during repass treatment.

Samples	Laser Power During Tape Laying (Repass Treatment)/W	Forming Speed/mm·min^−1^	Consolidation Force/N	Roller Material
NR	80 (80)	100	103	Silicon
1R	80 (80)	100	103	Silicon
2R	80 (80)	100	103	Silicon
3R	80 (80)	100	103	Silicon

**Table 2:** Surface roughness results of all groups of laminates. 10 and 16 are the number of prepreg layers.

Samples	$R_{a}$ (µm)	
	Present Study $\mathrm{CF}/\mathrm{PEKK}\;[0{}^{\circ}{]}_{10}$	Chanteli A et al. [19] $\mathrm{CF}/\mathrm{PEEK}\;[0{}^{\circ}{]}_{16}$
NR	4.02	4.88
1R	2.29	2.36
2R	1.74	2.12
3R	1.35	2.02

**Table 3:** Average ILSS of all groups of laminates.

Samples	ILSS (MPa)
NR	37.66 ± 7.72
1R	42.16 ± 5.15
2R	44.44 ± 6.21
3R	58.40 ± 3.11

**Table 4:** Average tensile strength of all groups of laminates.

Samples	Tensile Strength (MPa)
NR	745.11 ± 140.51
1R	956.63 ± 144.87
2R	1029.87 ± 123.24
3R	1054.75 ± 114.48

## Data Availability

The data presented in this study are openly available in FigShare at https://doi.org/10.6084/m9.figshare.28106873.
